# Development of a Cardiovascular Simulator for Studying Pulse Diagnosis Mechanisms

**DOI:** 10.1155/2017/6790292

**Published:** 2017-11-15

**Authors:** Min Jang, Min-Woo Lee, Jaeuk U. Kim, See-Yoon Seo, Sang-Hoon Shin

**Affiliations:** ^1^Department of East-West Medical Engineering, Sangji University, Wonju 26339, Republic of Korea; ^2^Department of Oriental Biomedical Engineering, Sangji University, Wonju 26339, Republic of Korea; ^3^Korea Institute of Oriental Medicine, Daejeon 305-811, Republic of Korea; ^4^Department of Korean Medicine, Sangji University, Wonju, Republic of Korea

## Abstract

This research was undertaken to develop a cardiovascular simulator for use in the study of pulse diagnosis. The physical (i.e., pulse wave transmission and reflection) and physiological (i.e., systolic and diastolic pressure, pulse pressure, and mean pressure) characteristics of the radial pulse wave were reproduced by our simulator. The simulator consisted of an arterial component and a pulse-generating component. Computer simulation was used to simplify the arterial component while maintaining the elastic modulus and artery size. To improve the reflected wave characteristics, a palmar arch was incorporated within the simulator. The simulated radial pulse showed good agreement with clinical data.

## 1. Introduction

Throughout history, pulse diagnosis has been a diagnostic technique used in Oriental medicine to yield many clinically significant results. The technique provides a way to monitor the overall health of the body by sensing the radial artery pulse.

To modernize pulse diagnosis, the mechanisms of pulse diagnosis need to be explained in terms of modern science. Previous studies in this area [1–3] can be classified into three categories: clinical studies, mathematical simulation studies, and physical simulator studies. Conducting clinical research is expensive and time-consuming, and manipulating biological variables is very challenging [4]. Mathematical modeling may also be impractical in some situations (e.g., arterial shunt) due to the assumptions made in the models. On the other hand, cardiovascular simulations are inexpensive and variables can be easily controlled using computer programs. Therefore, of the various types of research methods, research involving a cardiovascular simulator can be effective, minimize the time and money required for the study, and solve other practical issues [5].

Conventional simulators have been developed to perform functional evaluations of medical devices, such as left ventricular assist devices and artificial organs; thus, they are mainly focused on cardiac characteristics [6, 7]. Since the physical properties of arteries are important in pulse diagnosis research, heart-oriented cardiovascular simulators are inappropriate for investigations of pulse diagnosis.

Some studies have used simulators that focus on arterial characteristics. For example, Pahlevan and Gharib [8] researched the effect of aorta tapering on a physical heart model, and Knierbein et al. [9] developed an arterial tube model using one mother branch divided into two daughter branches. However, the tube did not include the radial artery, which is critical for pulse diagnosis. Full-branch elastic tube models have also been used to model the cardiovascular system [10, 11]. In humans, the elastic modulus of arteries increases as it reaches the periphery, but these simulators did not reflect the central-to-peripheral elastic modulus gradient.

Because pulse diagnosis measures the radial artery pulse wave, this study aimed to develop a cardiovascular simulator that focused on the radial pulse. A key requirement for investigating pulse diagnosis is the ability to replicate pulse wave transmission and the superposition of the forward and reflected waves. Additionally, the characteristics of the simulated pulse wave should be within the physiological range.

## 2. Method

The pulse wave at the radial artery is the superposition of the forward wave, produced by the heart, and the reflected wave from the aortic bifurcation at the iliac artery [15]. The factors affecting pulse wave velocity include the geometric properties and elastic modulus of the relevant arteries. Additionally, the aortic bifurcation also impacts pulse wave reflection. In this study, the simulated arteries were designed to have human arterial characteristics. However, human arterial trees are so complicated that simplification was needed. Thus, computer simulation was used to simplify the arterial tree. To improve the characteristics of the reflected wave, a shunt (palmar arch) was incorporated into the simulator. The radial pulse of the simulator was compared with that obtained from clinical data.

### 2.1. Arterial System Simplification

#### 2.1.1. Hemodynamic Analysis Program

**Figure 1:**
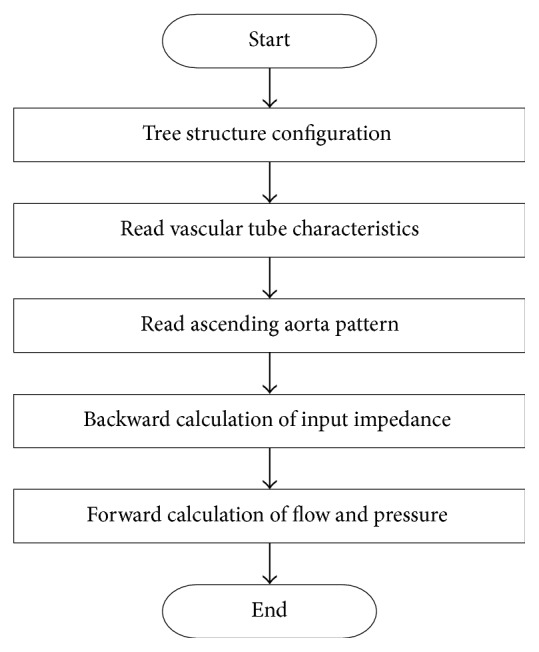
Computational algorithm for hemodynamics [12].

**Figure 2:**
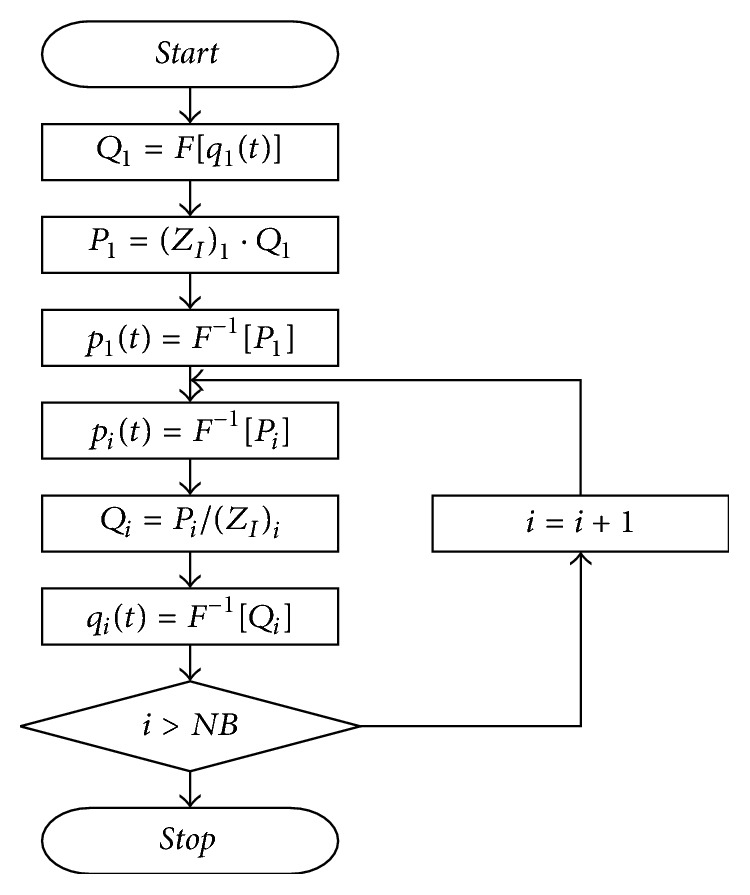
Computational algorithm for the calculation of the pressure and the flow in the artery [12].

Figure 1 shows the hemodynamic computational algorithm. After the arterial branch structure was defined, the arterial characteristics (e.g., length, elastic modulus, thickness, and radius) were input [12]. The input impedance was calculated as that between the peripheral artery and the ascending artery. After calculating the input impedance, blood pressure and blood flow were calculated from the ascending aorta to the peripheral artery using the measured flow or blood pressure patterns, as appropriate, at the ascending aorta [12]. Each branch was numbered for identification in the analysis, starting from the ascending aorta and continuing to the peripheral arteries. For this study, human vascular dimensions and elastic constants were obtained from the literature [13]. The input impedance (*Z*_*I*_) was defined as

(1) $$\begin{array}{c} Z_{I}=Z_{C}\frac{1+\Gamma e^{-2\gamma L}}{1-\Gamma e^{-2\gamma L}}, \end{array}$$

where Γ is the reflection constant, *γ* is the wave propagation constant, and *Z*_*C*_ is an impedance characteristic that was defined as

(2) $$\begin{array}{c} Z_{C}=\frac{\rho c_{0}}{A\sqrt{1-\sigma^{2}}}{\left(\;1-F_{10}\;\right)}^{-1/2}\left(\;\cos\frac{\phi }{2}+j\sin\frac{\phi }{2}\;\right), \end{array}$$

where *ρ* is the fluid density in a tube, *c*_0_ is the pulse wave velocity in an inviscid fluid, *A* is the cross-sectional area of the tube, *σ* is Poisson's ratio of the arterial wall, *F*_10_ is the Womersley function [16], and *ϕ* represents the phase lead of pressure in relation to wall displacement [13]. Blood pressures and blood flows were calculated in every branch from the ascending aorta to the peripheral artery. The calculation process is shown in Figure 2.

*q*_1_(*t*) is the given time-domain flow pattern in the ascending aorta, *p*_1_(*t*) is the *p* time-domain pattern in the ascending aorta, *F*[  ] is a Fourier transformation, *F*^−1^[  ] is an inverse Fourier transformation, and NB is the number of branches.

#### 2.1.2. Arterial System Simplification Using Simulation

**Figure 3:**
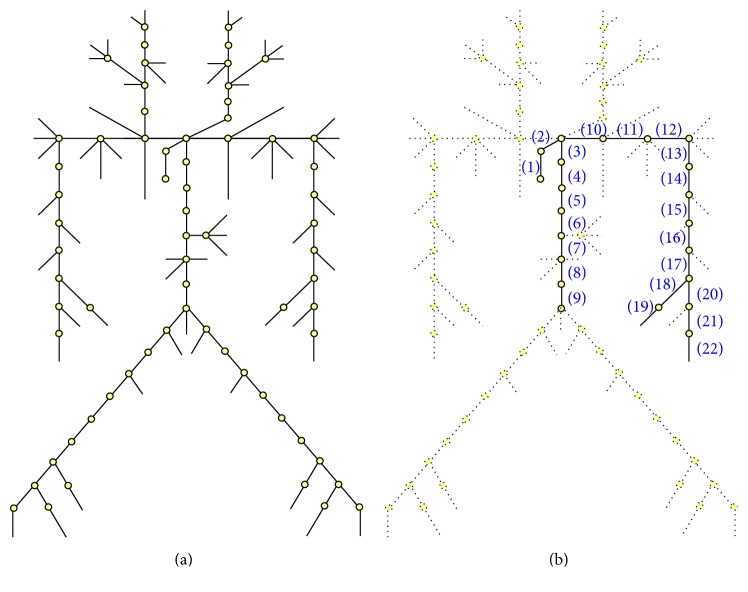
Simplification of the arterial tree. (a) Full-branch model [13], (b) simplified model.

**Figure 4:**
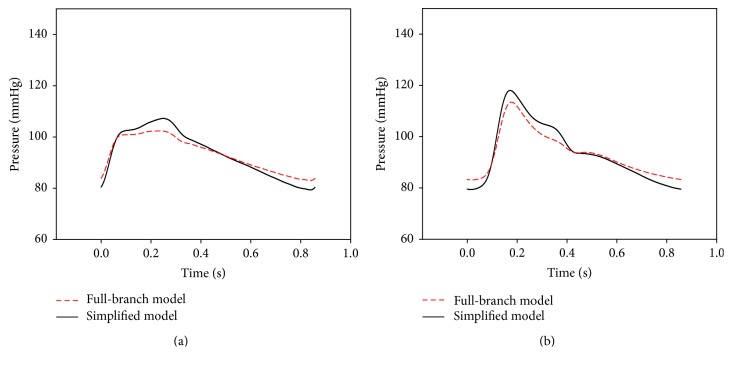
Comparisons of arterial tree models. (a) Ascending aorta, (b) radial artery.

The arterial properties described by Avolio [13] were used in this study. Some arterial branches are too complex and have too small diameters to be included in the simulator. The arterial tree was simplified using a hemodynamic analysis simulation (Figure 3).  Figure 4 shows a comparison between the two arterial tree models; the anatomical names of the arteries are shown in Table 1. With the same stroke volume input into both models, the simplified arterial model showed a mean pressure increase compared with that in the full-branch arterial model. However, the pulse waves did not change. Although the size of the reflected wave decreased slightly, the overall structure of the reflected wave was retained.

### 2.2. Cardiovascular Simulator

#### 2.2.1. System Composition

**Figure 5:**
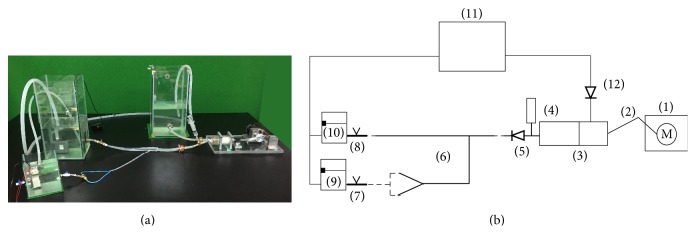
Simulator. (a) Photograph, (b) schematic diagram.

The composition of the cardiovascular simulator is shown in Figure 5. The simulator is composed of a pulse-generating component and an arterial component. The pulse-generating component consisted of a rotating motor (1), slider-crank (2), cylinder-piston (3), heart compliance chamber (4), and check valve ((5), (12)). The arterial component consisted of arteries (6), peripheral resistance ((7), (8)), and a reservoir ((9), (10), and (11)). The rotation of the motor was transformed into linear motion by the slider-crank mechanism, producing a pulsatile flow at the piston-cylinder. The fluid in the arterial system was drained to the reservoir, flowing back to the pulse-generating component.

A stepping motor (A200K-M599-G10, Autonics) was used for the rotating motor. A healthy heart has a normal cardiac output of approximately 5.3 L/min [17]. Given that the size of the simplified model used in this study was 55% of the full-branch arterial model, the cardiac output was set at 2.9 L/min. Therefore, the pulse-generating component was set at 82.8 bpm (heart rate) with a 35 mL stroke volume. To mimic blood viscosity, the working fluid was a mixture of 37% glycerin and 63% water [18]. The pressure sensor was an invasive type of pressure sensor (1620 pressure sensor, MSI Sensors), and data were acquired at a sampling rate of 1000 Hz. An ultrasound sensor (Bidop ES-100V3, Hadeco) was used to measure blood flow, and data were acquired at a sampling rate of 100 Hz. A laser distance-sensor (DT20-N244B, SICK) was used to measure the linear motion of the piston. All data were gathered and synchronized using a NI-DAQ (NI USB-6008, National Instruments).

#### 2.2.2. Arterial Model Construction and Simulation

**Figure 6:**
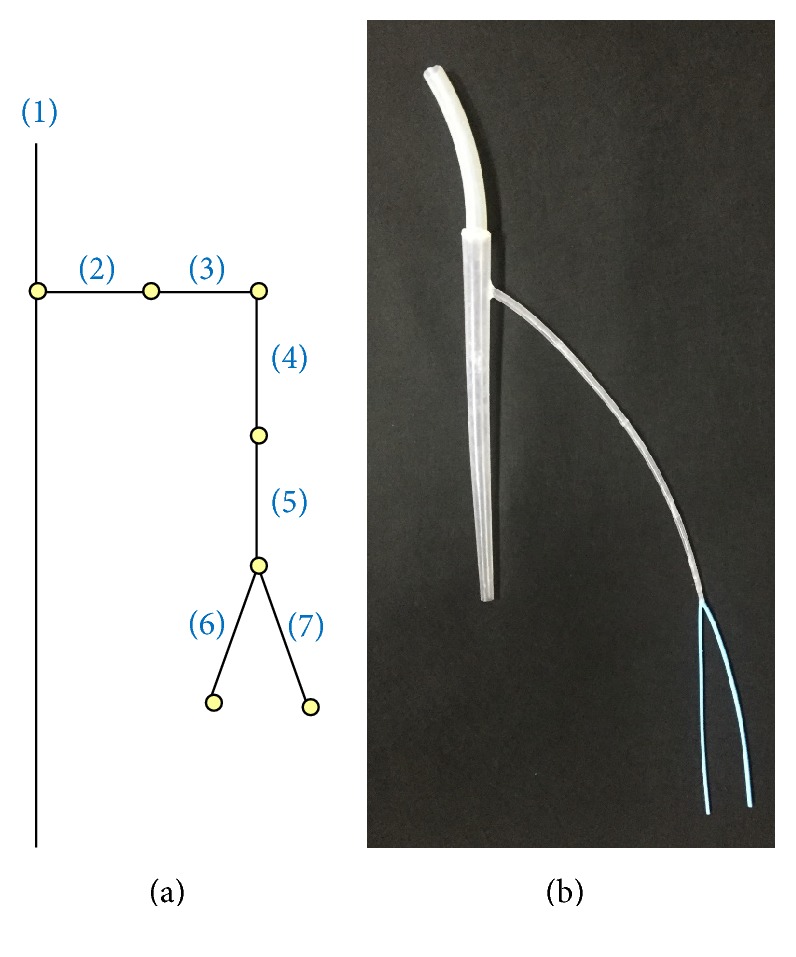
Arterial tree. (a) Schematic model, (b) photograph.

**Figure 7:**
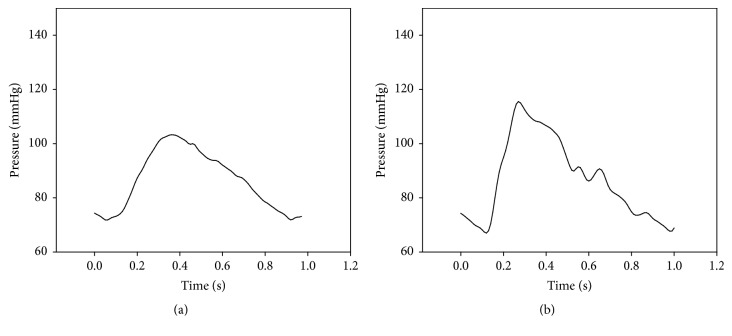
Simulated pressure wave. (a) Ascending aorta, (b) radial artery.

A simplified arterial model was also manufactured. The length, thickness, diameter, and elastic modulus of each artery were individually determined following the guidance of Avolio [13]. The elastic modulus of one model artery was 4 × 10^6^ dyne/cm^2^, and it was made of silicon (Sorta Clear 40, Smooth-On) mixed with a hardener. Another model artery had an elastic modulus of 8 × 10^6^ dyne/cm^2^, and it was also made of silicon (Smooth Sil 950, Smooth-On) mixed with a hardener.  Figure 6 shows the developed arterial model; the vessel properties are shown in Table 2.  Figure 7 shows the waveform results generated by the simulator.

#### 2.2.3. Arterial Tree Improvement

**Figure 8:**
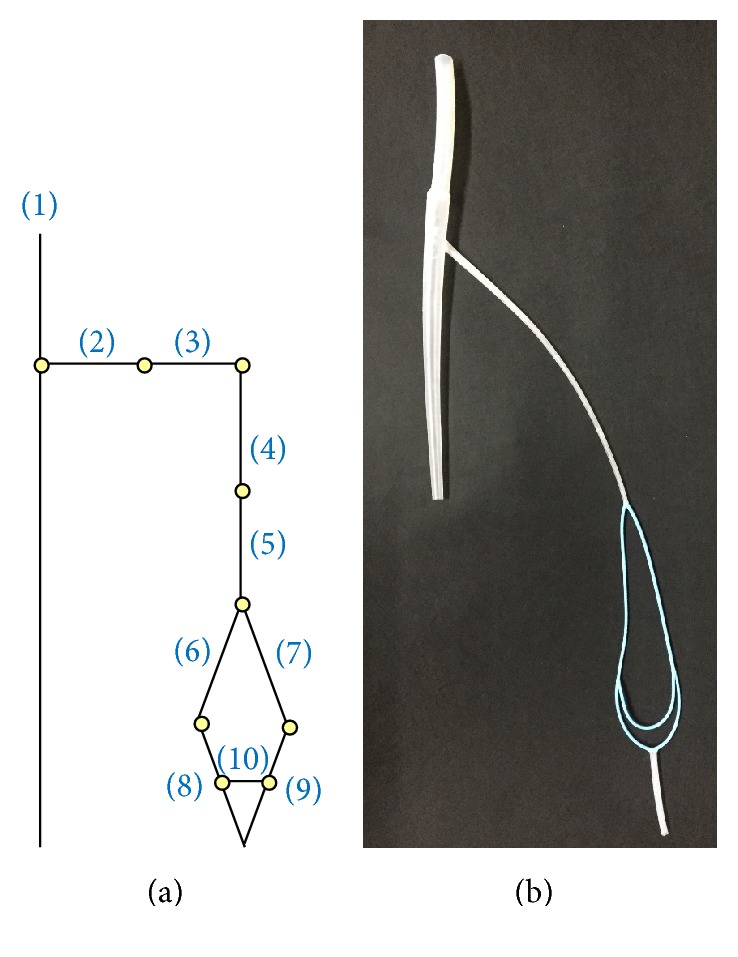
Improvement of arterial tree (with shunt). (a) Schematic model, (b) photograph.

Figure 7(b) shows the radial artery pressure wave. However, the waveform is different from a clinically measured radial waveform because of the superposition of the reflected wave from the end of the radial artery. There is a shunt connecting the radial artery to the ulnar artery [19, 20].  Figure 8 shows arterial tree with the shunt, and the properties of the arteries are shown in Table 3.

## 3. Results and Discussion

**Figure 9:**
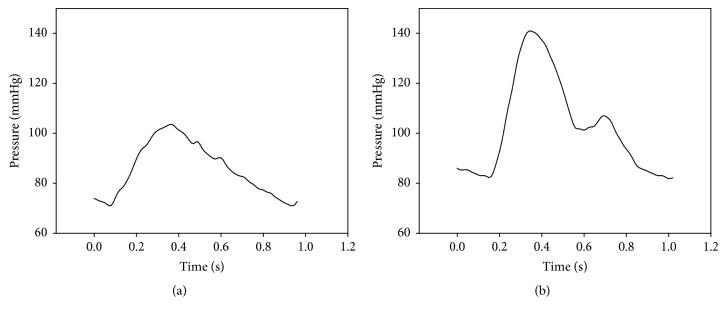
Simulator results using a simplified arterial model. (a) Ascending aorta, (b) radial artery.

**Figure 10:**
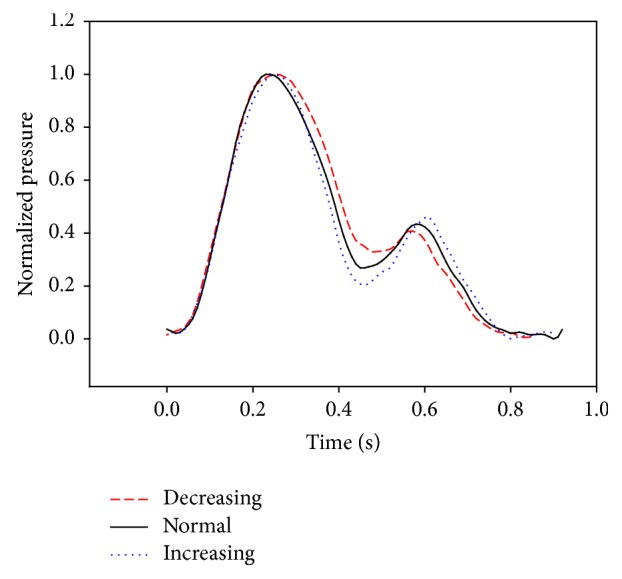
The effects of the reflection ratio of the aortic bifurcation to the radial artery.

**Figure 11:**
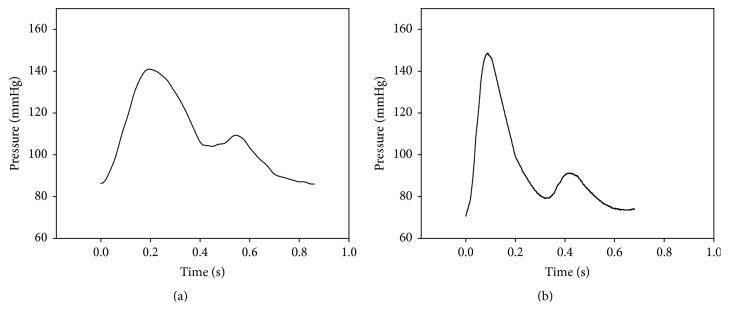
Comparison of the radial pulse. (a) Simulator, (b) clinical data [14].

Figures 9, 10, and 11 show the simulator results using the simplified arterial model. Specifically, Figure 9 shows the pressure wave measured simultaneously at the ascending aorta and the radial artery.  Figure 10 shows how changes in peripheral resistance affect the pressure at the radial artery as well as the effects of the reflection ratio from the aortic bifurcation to the radial artery.  Figure 11 shows a comparison of the pressure wave from the simulator with clinical data [14].

The cardiovascular simulator was developed to satisfy the following three conditions: (1) a focus on the radial pulse, (2) a pulse wave produced with superposition of the forward wave from the heart and the reflected wave from the aortic bifurcation, and (3) a pressure wave within the physiological range.

The properties of young arteries were used in the simulator [13].  Figure 7 shows the simulator results in the absence of a shunt. In the figure, a radial artery pressure wave shows a sharp peak at the beginning of systole. Because the end of the radial artery is closest to the measuring point, the reflected wave from the end of the radial artery comes first. However, this does not reflect the physiological situation. To reduce this discrepancy between the model and actual physiology, a shunt was incorporated into the simulation.  Figure 7 shows results before adding the shunt and Figure 9 shows results after adding the shunt. The pressure wave in Figure 9 shows that the sharp peak in early systole had disappeared, resulting in a waveform similar to what might be seen for a young person [14]. The shunt adaptation allowed dissemination of the peripheral reflection, solving the artefact observed in Figure 7.

The distance between the pulse-generating component and the measurement site at the ascending aorta was 4 cm, and 63 cm from that at the aortic bifurcation. Differences in the forward wave arrival time and the difference in locations are seen in Figure 9. Pulse wave velocity (PWV) was calculated using a foot-to-foot method and found to be 5.9 m/s [21], which is within the clinical range of 5–8 m/s [22]. The elastic modulus used for the simulator was based on data from a young individual; therefore, the PWV was less than that associated with adults [23]. The superposition time was then calculated to be 0.13 s; the incisura point is shown in Figure 9. According to Figure 9, the superimposition of the reflected wave occurred after the incisura point.

In the pulse wave, the first peak indicates the maximum value of the forward wave, and the second peak indicates the maximum value of the reflected wave. By comparing the difference in time between the two peaks, the gap between the first and second peak times in the ascending aorta was closer than that for the radial artery. This was due to the faster superposition time at the ascending aorta than at the radial artery [14]. Although the reflected wave traveled 129.8 cm to the radial artery, it traveled only 74.8 cm to the ascending aorta, resulting in the time difference. Therefore, the reflected wave shown in the radial artery was concluded to have come from the aortic bifurcation, as was demonstrated experimentally.

Figure 10 shows the pulse wave of the radial wave with different levels of aortic periphery resistance. With increasing resistance, an increase in mean pressure and a decrease in pulse pressure were observed. To compare the sizes of the forward and reflected waves, the measured pressure waveforms were normalized. As a result, as aortic periphery resistance increased, there was an increase in the gap between the incisura point and the second peak. This shows that the increase in the reflected wave was larger than that in the forward wave. The waveform and the location of the reflection remained unchanged, meaning that aortic peripheral resistance was only increased in the reflected wave. An animal experiment conducted by Alastruey et al. [24] also demonstrated that an increase in peripheral resistance accounted for the increase in the reflected wave. Thus, the developed simulator replicated the superposition of the forward wave of the pulse-generating component and the reflected wave of the aortic bifurcation.

The pressure wave of the ascending aorta at the simulator was 71.0–103.0 mmHg, similar to clinically acquired data (73.0–116.0 mmHg). Similarly, the pressure wave at the radial artery was 86–140.9 mmHg, compared with 70.0–148.0 mmHg for clinical data, indicating that the simulated data were within the data range for a healthy person [14]. As the pulse wave traveled from the ascending aorta to the radial artery, the pulse pressure at the simulator increased by 22.3 mmHg (range, 2.6–54.9 mmHg); in a clinical example, the pulse pressure increased by 25 mmHg (range, 43.0–78.0 mmHg). An increase in the pulse pressure may be explained by a decreased arterial diameter and an increased elastic modulus, leading to increased systolic pressure [25]. This phenomenon was also shown in the developed simulator.

This study had a few limitations. There are many studies regarding the geometric structure of the palmar arch, which was developed at the distal end of the radial artery, in this study. However, the anatomical parameters such as the length, internal diameter, thickness, and Young's modulus were difficult to determine. We expect that the simulator can be enhanced by determining precise boundary conditions using more accurate parameters for the palmar arch artery.

## 4. Conclusions

In this study, a cardiovascular simulator was developed for use in studying the mechanisms associated with pulse diagnoses. The radial pulse from the simulator showed superposition of the forward wave from the heart and the backward wave from the aortic bifurcation. Additionally, the generated pulse wave was within the physiological ranges. Thus, this cardiovascular simulator may be useful for future pulse diagnosis studies.

## Figures and Tables

**Table 1:** Anatomical names of the simplified arterial tree.

Branch number	Anatomical name
(1)	Ascending aorta
(2)	Aortic arch 1
(3)	Aortic arch 2
(4)	Thoracic aorta 1
(5)	Thoracic aorta 2
(6)	Thoracic aorta 3
(7)	Abdominal aorta 1
(8)	Abdominal aorta 2
(9)	Abdominal aorta 3
(10)	Subclavian artery 1
(11)	Subclavian artery 2
(12)	Axillary artery 1
(13)	Axillary artery 2
(14)	Brachial artery 1
(15)	Brachial artery 2
(16)	Brachial artery 1
(17)	Brachial artery 2
(18)	Radial artery 1
(19)	Radial artery 2
(20)	Ulnar artery 1
(21)	Ulnar artery 2
(22)	Ulnar artery 3

**Table 2:** Parameters used in the arterial tree.

Number	Branch	Thickness_in (cm)	Thickness_out (cm)	Radius_in (cm)	Radius_out (cm)	Length (cm)	Elastic modulus (×10^6^ dyne/cm^2^)
(1)	Aorta	0.16	0.05	1.47	0.58	41.40	4
(2)	L. subclavian_1	0.08	0.07	0.44	0.40	10.70	4
(3)	L. subclavian_2	0.07	0.06	0.40	0.33	10.60	4
(4)	L. subclavian_3	0.06	0.05	0.33	0.33	8.80	4
(5)	L. subclavian_4	0.05	0.04	0.33	0.33	9.00	4
(6)	L. radial	0.04	0.03	0.20	0.20	27.20	8
(7)	L. ulnar	0.04	0.03	0.22	0.22	26.90	8

L, left.

**Table 3:** Parameters used in the arterial tree (with palmar arch).

Number	Branch	Thickness_in (cm)	Thickness_out (cm)	Radius_in (cm)	Radius_out (cm)	Length (cm)	Elastic modulus (×10^6^ dyne/cm^2^)
(1)	Aorta	0.16	0.05	1.47	0.58	41.40	4
(2)	L. subclavian_1	0.08	0.07	0.44	0.40	10.70	4
(3)	L. subclavian_2	0.07	0.06	0.34	0.33	10.60	4
(4)	L. subclavian_3	0.06	0.05	0.33	0.33	8.80	4
(5)	L. subclavian_4	0.05	0.04	0.33	0.33	9.00	4
(6)	L. radial	0.04	0.03	0.20	0.20	27.20	8
(7)	L. ulnar	0.04	0.03	0.22	0.22	26.90	8
(8)	Deep palmar arch_1	0.03	0.02	0.69	0.69	14.00	8
(9)	Deep palmar arch_2	0.03	0.02	0.22	0.22	14.00	8
(10)	Superficial palmar arch	0.03	0.02	0.21	0.21	14.00	8

L, left.
